# Genome Sequences of Mycobacteriophages Kerberos, Pomar16, and StarStuff

**DOI:** 10.1128/genomeA.00690-17

**Published:** 2017-08-10

**Authors:** Deborah Jacobs-Sera, Oana Catinas, Mariceli Fernandez-Martinez, Amelia Garcia, Rebecca A. Garlena, Carlos A. Guerrero Bustamante, Michelle H. Larsen, Rosa H. Medellin, Martin Y. Melendez-Ortiz, Crystal M. Melendez-Rivera, Alondra K. Mercado-Andino, Abner J. Mercado-Delgado, Cathia P. Ortiz-Ortiz, Ana M. Quesada-Gordillo, Jacqueline M. Ramos, Michael R. Rubin, Daniel A. Russell, Rachna A. Sadana, Sanghamitra Saha, Edwin Vazquez, David Villarreal, Graham F. Hatfull

**Affiliations:** a Department of Biological Sciences, University of Pittsburgh, Pittsburgh, Pennsylvania, USA; b Africa Health Research Institute, Durban, South Africa; c Department of Biology, University of Puerto Rico at Cayey, Cayey, Puerto Rico, USA; d Department of Natural Sciences, University of Houston–Downtown, Houston, Texas, USA; e Department of Medicine, Albert Einstein College of Medicine, New York, New York, USA

## Abstract

We describe the genome sequences of three closely related mycobacteriophages, Kerberos, Pomar16, and StarStuff, isolated at similar times but from geographically distinct regions. All three genomes are similar to those of other subcluster A2 phages, such as L5 and D29, are temperate, and have siphoviral virion morphologies.

## GENOME ANNOUNCEMENT

A large collection of sequenced mycobacteriophages—phages that infect mycobacterial hosts—reveals them to span a spectrum of genetic diversity (1). They can be grouped into clusters (some of which are divided into subclusters) and singletons according to their overall relatedness (2), and the collection of over 1,300 sequenced phages currently spans 26 clusters and 6 singletons (http://phagesdb.org). Most of these phages were isolated on a single host strain (*Mycobacterium smegmatis* mc^2^155), and approximately 10% of the phages efficiently infect *Mycobacterium tuberculosis* mc^2^7000. For some other phages, host range expansion mutants that efficiently infect *M. tuberculosis* can be isolated (3). Those phages that efficiently infect *M. tuberculosis* map within subclusters A2 and A3 and all subclusters within cluster K (3). Mycobacteriophages not only have provided insights into phage diversity and evolution but also have been exploited for various tools and applications (4), including the use of D29 in a rapid amplification strategy for tuberculosis diagnosis (5).

In 2015, phages Kerberos, Pomar16, and StarStuff were isolated on *M. smegmatis* mc^2^155 using soil samples and an enrichment procedure. The samples were collected in geographically distinct regions, Kerberos from Houston, TX, Pomar16 from Aibonito, PR, and StarStuff from Pinetown, South Africa. Following plaque purification and amplification, DNA was isolated and sequenced using Illumina MiSeq 150-bp single-end runs. Trimmed reads were assembled using Newbler, and single contigs were assembled. Genome lengths were 52,753 bp, 52,833 bp, and 52,785 bp, and read coverages were 506, 564, and 3,354 for Kerberos, Pomar16, and StarStuff, respectively. All three phages have defined ends with 10-base 3′ single-stranded DNA extensions (5′-CGGTCGGTTA), and all are approximately 63.5% G+C. Electron microscopy shows that all three phages have siphoviral morphologies with icosahedral heads approximately 55 nm in diameter and flexible noncontractile tails approximately 110 nm long.

All three genomes were annotated using DNA Master (http://cobamide2.bio.pitt.edu/), Glimmer (6), GeneMark (7), Aragorn (8), tRNAscan-SE (9), BLASTP (10), HHPred (11), and Phamerator (12). BlastN comparisons showed that the three genomes are very closely related to each other and have greater than 98% nucleotide identity across their entire genome spans. Each genome contains 93 protein-coding genes and 5 tRNA genes. Their overall genome architectures are similar to those of other subcluster A2 phages, including L5 and D29 (13, 14), with rightward-transcribed virion structure and assembly genes in the left arms and leftward-transcribed nonstructural genes in the right arms. All encode a putative repressor protein with similarity to the L5 repressor (78% amino acid identity). The integration systems are closely related to those of D29 and are predicted to use the same *attB* site for integration.

The genome most closely related to Kerberos, Pomar16, and StarStuff is phage D29, which was previously shown to contain a 3.6-kbp deletion when aligned to phage L5 (14). Thus, all three genomes are likely to be very close relatives of the putative temperate parent of D29.

### Accession number(s).

Pomar16, Kerberos, and StarStuff are available at GenBank with accession numbers KX574455, KX758538, and KX897981, respectively.
